# Supplementary material for: The localization of centromere protein A is conserved among tissues
Source: Commun Biol. 2023 Sep 21;6:963. doi: 10.1038/s42003-023-05335-7 (PMC10514049; doi:10.1038/s42003-023-05335-7)
Supplement: Supplementary file 2 — Supplementary material [file 42003_2023_5335_MOESM2_ESM.pdf]

**Supplementary Material for**

# The localization of centromere protein a is conserved among tissues

Eleonora Cappelletti[#], Francesca M. Piras[#], Lorenzo Sola, Marco Santagostino, Jessica L. Petersen, Rebecca R. Bellone, Carrie J. Finno, Sichong Peng, Ted S. Kalbfleisch, Ernest Bailey, Solomon G. Nergadze, Elena Giulotto[*]

[*] Corresponding author, e-mail: elena.giulotto@unipv.it
[#] These authors contributed equally

## Table of Contents

**Supplementary Figure 1:** Mapping quality of aligned Illumina and PacBio reads in the centromeric region of chromosome 11 from EquCab3.0 and EquCab3.0_cen references.

**Supplementary Figure 2:** CENP-A enriched domains on chromosome 11 in the fibroblast cell line and in the four tissues from the FAANG horses.

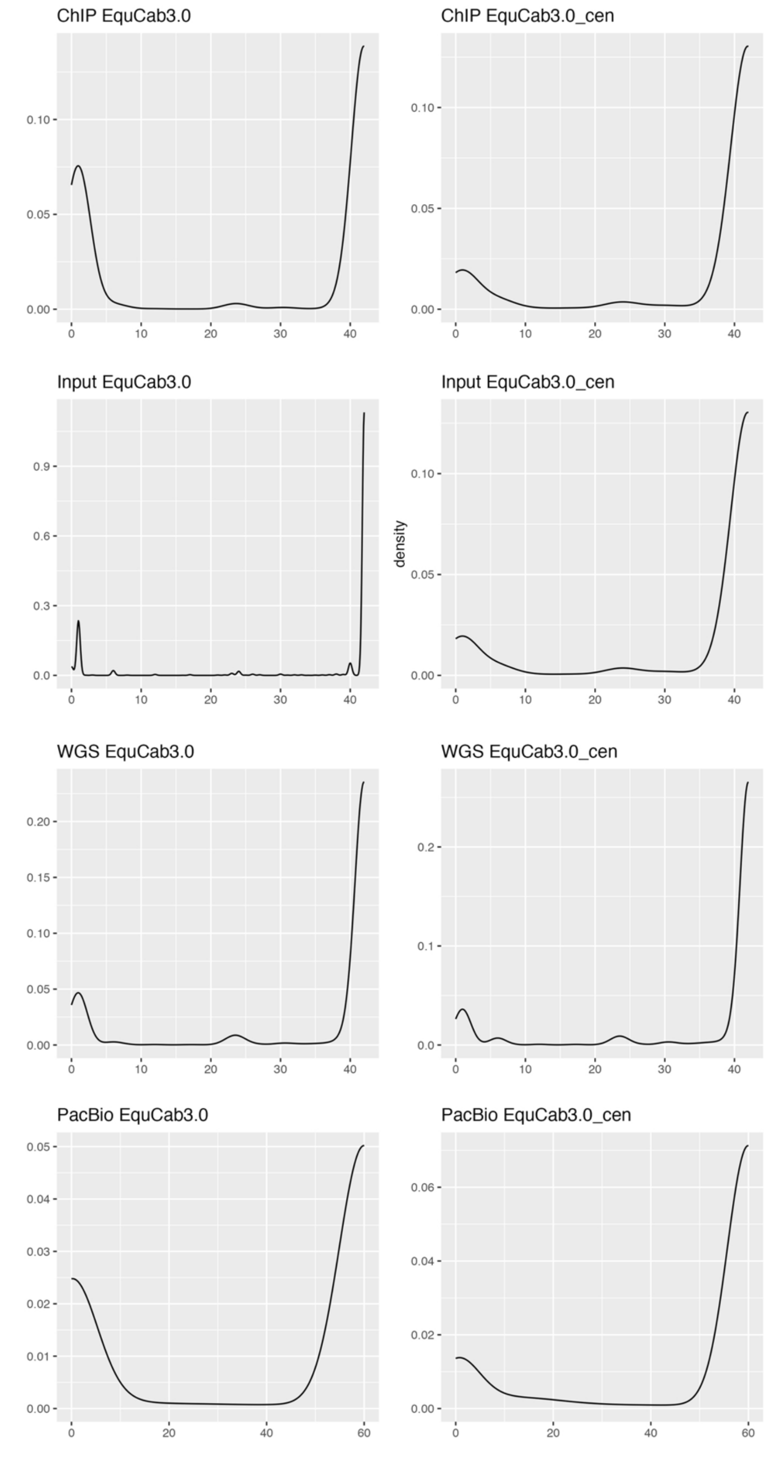


**Supplementary Figure 1: Mapping quality of aligned Illumina and PacBio reads in the centromeric region of chromosome 11 from EquCab3.0 and EquCab3.0_cen references.** Density plots showing mapping quality of ChIP reads, Input reads, Illumina WGS reads and PacBio reads of Twilight aligned in the centromeric region of chromosome 11 in the EquCab3.0 (left) and in the EquCab3.0_cen (right) assemblies. The y axis reports the density. The x axis reports mapping quality.

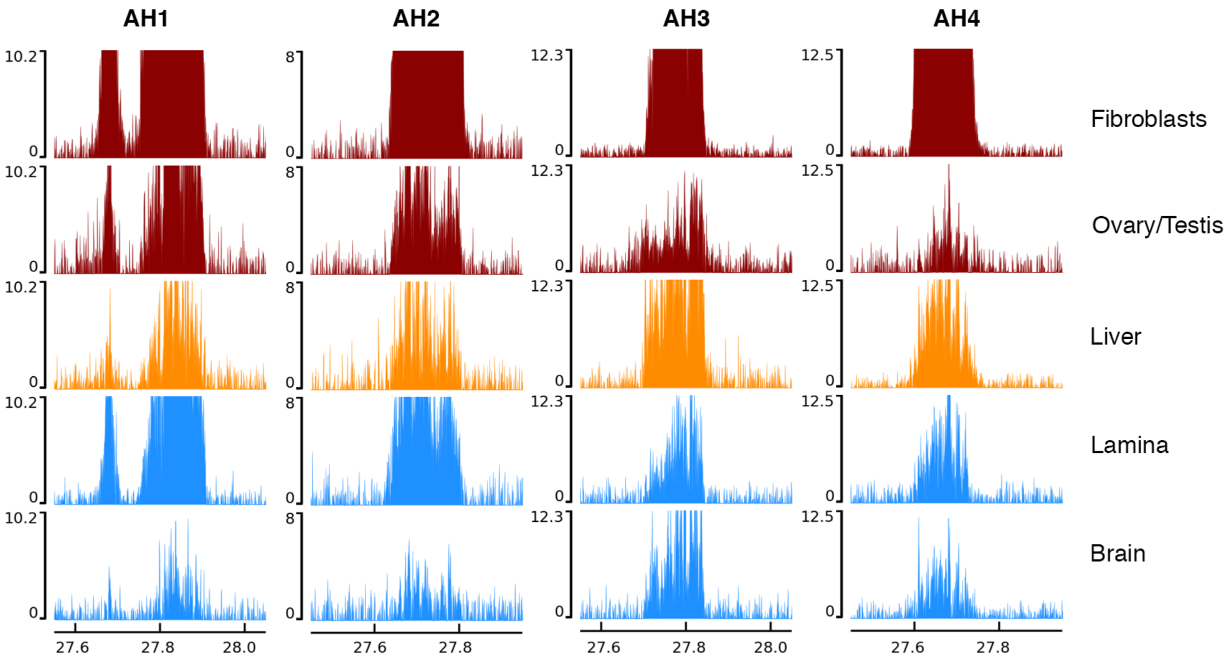


**Supplementary Figure 2: CENP-A enriched domains on chromosome 11 in the fibroblast cell line and in the four tissues from the FAANG horses.** ChIP-seq profiles of the CENP-A enriched domains. The y-axis reports the normalized read counts whereas the x-axis reports the coordinates on the EquCab3.0_cen reference genome. For each horse, the scale of the peak with the lowest enrichment was used to plot all enrichment peaks. Color code refers to the embryonic origin: mesodermal (red), endodermal (yellow), ectodermal (cyan).
